# Metabolic profiling and expression analysis of key genetic factors in the biosynthetic pathways of antioxidant metabolites in mungbean sprouts

**DOI:** 10.3389/fpls.2023.1207940

**Published:** 2023-06-16

**Authors:** Byeong Cheol Kim, Insu Lim, Jungmin Ha

**Affiliations:** ^1^ Department of Plant Science, Gangneung-Wonju National University, Gangneung, Republic of Korea; ^2^ Haeram Institute of Bakery Science, Gangneung-Wonju National University, Gangneung, Republic of Korea

**Keywords:** mungbean sprout, UPLC, metabolic profiling, gene expression, antioxidants

## Abstract

Mungbeans (*Vigna radiata* L.), a major legume crop in Asia, contain higher amounts of functional substances than other legumes, such as catechin, chlorogenic acid, and vitexin. Germination can improve the nutritional value of legume seeds. Here, 20 functional substances were profiled in germinated mungbeans and the expression levels of the transcripts of key enzymes in targeted secondary metabolite biosynthetic pathways were identified. VC1973A, a reference mungbean elite cultivar, had the highest amount of gallic acid (99.93 ± 0.13 mg/100 g DW) but showed lower contents of most metabolites than the other genotypes. Wild mungbeans contained a large amount of isoflavones compared with cultivated genotypes, especially for daidzin, genistin and glycitin. The expression of key genes involved in biosynthetic pathways had significant positive or negative correlations with the target secondary metabolite contents. The results indicate that functional substance contents are regulated at the transcriptional level, which can be applied to improve the nutritional value of mungbean sprouts in molecular breeding or genetic engineering, and wild mungbeans are a useful resource to improve the quality of mungbean sprouts.

## 1 Introduction

Secondary metabolites in plants, including phenolic compounds and flavonoids, are produced through defense mechanisms against biotic and abiotic stresses (Hartmann, 2007; Zandalinas et al., 2017). These are known to have diverse physical and chemical traits, such as color, flavor, and medicinal effects, leading to high economic value (Pagare et al., 2015). Legume crops, such as soybean, mungbean (*Vigna radiata* L.), and pigeonpea (*Cajanus cajan*), were reported to contain large amounts of functional substances, including catechin, neo/chlorogenic acid, and iso/vitexin (Fu et al., 2008; Cao et al., 2011). Catechin and chlorogenic acid have been reported to have protective effects against colorectal cancers, reducing the incidence by 77–83% in humans (Lu et al., 2022). Vitexin also provides many health benefits including antioxidant, anti-inflammatory, antinociceptive, cardioprotective, and neuroprotective effects (He et al., 2016).

Mungbeans, one of the major legume crops in Asia, are cultivated on over six million hectares worldwide, with an annual grain production of three million tons (Gayacharan et al., 2020). Because of their high carbohydrate content (50–60%) and protein content (20–24%), mungbeans are an important nutritional source in many developing countries and are mostly consumed as starch grains or germinated sprouts (Peng et al., 2008; Tang et al., 2014). Ethanol extracts of mungbean seeds contain high levels of phenolic compounds, such as catechin, chlorogenic acid, and vitexin (An et al., 2020). Germination increases the nutritional value of the seeds (Hung et al., 2012). Germination can improve the functional and nutritional quality of legumes by increasing protein digestibility and reducing anti-nutritional factors, and germinated legumes can be consumed as protein supplements with functional agents (Kartikeyan et al., 2022). In mungbeans, the contents of functional substances, including carotenoids, vitamin E, and various phenolic compounds, increase after germination (Li et al., 2021). Mungbean sprouts grow rapidly indoors without being affected by the weather. Ethanol extracts of mungbean sprouts were reported to contain higher amounts of phenolic compounds than the seeds, even when compared with other legume crops (Peng et al., 2008). Therefore, mungbean sprouts are a healthy food that is easy to produce and can efficiently supply functional substances to the human diet.

The secondary metabolite contents in soybeans vary depending on the cultivar, cultivation period, and tissue (Yun et al., 2020). In cauliflowers (*Brassica aleracea* L. ssp. *botrytis*), secondary metabolites, such as anthocyanins, carotenoids, and phenolic compounds, were reported to vary according to genotypes with different floret colors (Park et al., 2013). Although the contents of secondary metabolites in mungbeans can vary depending on the cultivation period, genotype, and environmental conditions, these studies only examined a few major cultivars (Guo et al., 2012; An et al., 2020). Therefore, we hypothesized that the secondary metabolites would vary significantly among the 50 different mungbean genotypes and they would be regulated in the level of transcription. In this study, the metabolic profiling of 50 mungbean genotypes of diverse origins and phenotypes, including wild species, was performed using ultra-high-performance liquid chromatography (UPLC). Key genetic factors underlying the biosynthesis of secondary metabolites were identified. This study provides new insights into the value of mungbean sprouts as a nutritional resource with high functional substance contents.

## 2 Materials and methods

### 2.1 Sample preparation

All mungbean seeds were harvested at the Gangneung-Wonju National University Experimental Farm in Gangneung, South Korea (37.77°N, 128.86°E) in 2021. A total of 50 mungbean genotypes were germinated (**Supplementary Table 1**). Mungbean seeds were rinsed three times, and then soaked with distilled water in dark conditions at 37°C for 17 h using an incubator (JEIO TECH. ISS-4075R, Daejeon, Korea). The mungbean sprouts were cultivated using the water-spraying method in a sprout cultivator (Sundotcom, ST001A, Seoul, Korea). Water spraying was conducted for 2 mins every four hours at 30 ± 2°C for 3 days according to the methods of a previous study (Kim et al., 2021). Three-day-old mungbean sprouts were dried at 70°C for 24 h, and then ground into a fine powder (Gan et al., 2017). The samples were extracted at 0.1 g/mL (w/v) with 70% ethanol (EtOH) and stored at -20°C until further analysis.

### 2.2 UPLC-photodiode array method

UPLC was performed for metabolic profiling (Nexera series equipped with MPM-40, SCL-40, SPD-M40, LC-40, SIL-40, and CTO-40 units from Shimadzu, Kyoto, Japan) using a photodiode array (PDA) detector. Secondary metabolites were separated on a ZORBAX SB-C18 column (3.5 μm, 4.6 x 150 mm; Agilent, PN 863953-902, Santa Clara, USA). The mobile phase gradients of ultrapure water-0.1% acetic acid solution (v/v; solvent A) and acetonitrile (solvent B) flowed at 1 mL/min as follows: 0–10 min 95–90% A, 10–11 min 90–85% A, 11–15 min 85–80% A, 15–16 min 80–70% A, 16–25 min 70–65% A, 25–28 min 65–50% A, 28–32 min 50% A, 32.1 min solvent A was increased from 50 to 95%, and 32.1–40 min 95% A. The column oven temperature was set at 40°C, and the sample injection volume was 2 μL. The results were determined based on standard calibration curves (10–100 mg/L) with three replicates. The photon wavelength of the detector scan range was set between 190 and 800 nm. The chromatograms of metabolites, such as biochanin A (Chemfaces, CFN99734, Wuhan, China), catechin (Chemfaces, CFN99646), and genistin (Chemfaces, CFN90250) were extracted at 270 nm, while caffeic acid (Chemfaces, CFN99646), daidzein (Chemfaces, CFN98774), daidzin (Chemfaces, CFN99101), formononetin (Chemfaces, CFN99962), gallic acid (Chemfaces, CFN99624), genistein (Chemfaces, CFN98681), glycitein (Chemfaces, CFN99106), glycitin (Chemfaces, CFN99105), isovitexin (Chemfaces, CFN98620), neochlorogenic acid (Chemfaces, CFN97472), p-coumaric acid (Chemfaces, CFN98794), resveratrol (Chemfaces, CFN98791), syringic acid (Chemfaces, CFN98884), t-ferulic acid (Chemfaces, CFN92394), and vitexin (Chemfaces, CFN98601) were extracted at 280 nm, and chlorogenic acid (Chemfaces, CFN99116), coumestrol (Chemfaces, CFN96040), kaempferol (Chemfaces, CFN98838), myricetin (Chemfaces, CFN98877), and quercetin (Chemfaces, CFN99272) were extracted at 330 nm (**Table 1**; **Supplementary Figure 1**).

**Table 1:** List of UPLC standard materials.

No.	Name	Formula		Cas No.	Molecular weight	External ID	PubChem SID
**1**	Gallic acid	C7H6O5		149-91-7	170.1	C01424	4609
**2**	Neochlorogenic acid	C16H18O9		906-33-2	354.3	C17147	96023599
**3**	Catechin	C15H14O6		154-23-4	290.3	C06562	8791
**4**	Chlorogenic acid	C16H18O9		327-97-9	354.31	C00852	4109
**5**	Caffeic acid	C9H8O4		331-39-5	180.15	C01481	4652
**6**	Syringic acid	C9H10O5		530-57-4	198.2	C10833	13016
**7**	p-coumaric acid	C9H8O3		501-98-4	164.2	C00811	4069
**8**	Daidzin	C21H20O9		552-66-9	416.38	C10216	12402
**9**	Glycitin	C22H22O10		40246-10-4	446.41	C16195	47205503
**10**	t-Ferulic acid	C10H10O4		537-98-4	194.2	C01494	4664
**11**	Vitexin	C21H20O10		3681-93-4	432.4	C01460	4637
**12**	Isovitexin	C21H20O10		38953-85-4	432.4	C01714	4851
**13**	Genistin	C21H20O10		529-59-9	432.38	C09126	11318
**14**	Myricetin	C15H10O8		529-44-2	318.2	C10107	12293
**15**	Resveratrol	C14H12O3		501-36-0	228.2	C03582	6374
**16**	Daidzein	C15H10O4		486-66-8	254.2	C10208	12394
**17**	Glycitein	C16H12O5		40957-83-3	284.26	C14536	17395536
**18**	Quercetin	C15H10O7		117-39-5	302.2	C00389	3679
**19**	Genistein	C15H10O5		446-72-0	270.2	C06563	8792
**20**	Coumestrol	C15H8O5		479-13-0	268.2	C10205	12391
**21**	Kaempferol	C15H10O6		520-18-3	286.2	C05903	8191
**22**	Formononetin	C16H12O4		485-72-3	268.27	C00858	4115
**23**	Biochanin A	C16H12O5		491-80-5	284.26	C00814	4072

### 2.3 Total RNA extraction and quantitative reverse-transcription polymerase chain reaction

Total RNA was extracted with GeneAll Ribospin™ Plant Kit (Cat. 307-150; Gene all, Seoul, Korea). The extracted RNA was quantified using a UV/Vis Nanodrop spectrophotometer (MicroDigital, Nabi, Seongnam, Korea). RNA purity was measured using the absorbance ratio of OD 260/280 and OD 260/230. The cDNA library was synthesized using the PrimeScript™ RT reagent Kit with gDNA Eraser (RR047A; TaKaRa, Tokyo, Japan) according to the manufacturer’s protocol with 1 μg of RNA. The quantitative reverse-transcription polymerase chain reaction (qRT-PCR) was conducted using the TB Green® Premix Ex Taq™∥ Kit (Tli RNaseH Plus) (RR820A; TaKaRa, Tokyo, Japan). The PCR primer sequences are listed in **Supplementary Table 2**. qRT-PCR was performed using the 12 genotypes of which the secondary metabolite contents varied significantly.

### 2.4 Statistical analysis

The contents of the secondary metabolites are shown as the mean ± standard deviation from three replicates. Experimental data from the UPLC quantitative analysis were analyzed by a one-way analysis of variance (ANOVA), and a significance analysis was performed using Duncan’s Multiple Range test at 0.05 (Stahle and Wold, 1989). Multivariate statistical analysis of the secondary metabolite content detected in the 50 mungbean genotypes was performed using a principal component analysis (PCA) and hierarchical clustering (HCA). All data analyses were performed using RStudio (R Core Team, 2017). A Pearson correlation analysis between gene expression and metabolites was performed on eight out of 12 randomly selected genotypes.

## 3 Results

### 3.1 Identification of secondary metabolites in mungbean sprouts

**Figure 1:**
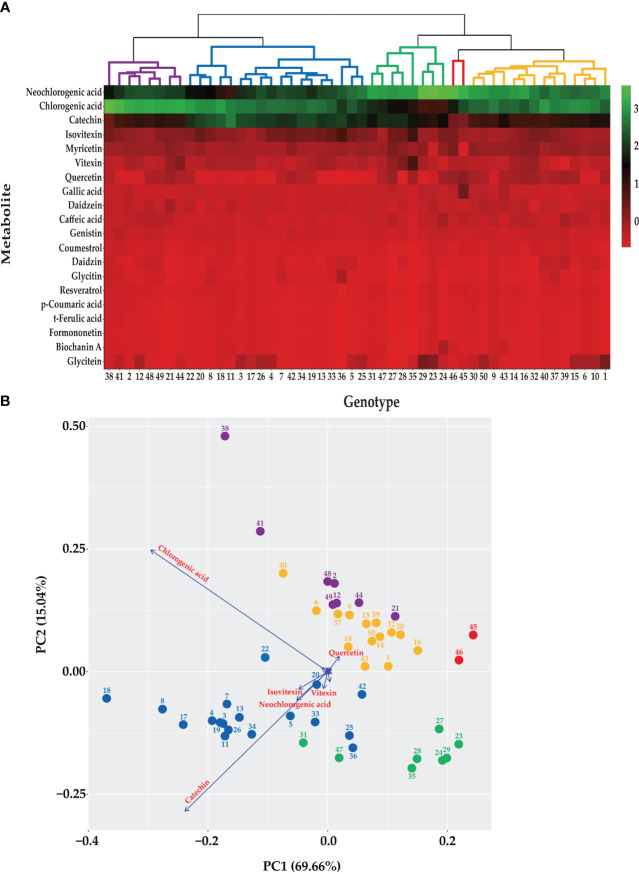
Metabolic profiling of 50 mungbean genotypes. **(A)** Heatmap 362 of the contents of the secondary metabolites in 50 mungbean genotypes. The contents of 20 secondary metabolites are indicates by color scale from red to green. Upper tree indicates clustering by HCA of 50 mungbean genotypes according to the contents of the secondary metabolites. **(B)** PCA of mungbean genotypes according to the contents of secondary metabolites. The circle colors indicate clustering results of mungbean genotypes according to HCA. Arrows indicate the major metabolites of mungbean sprouts according to UPLC analysis.

**Figure 2:**
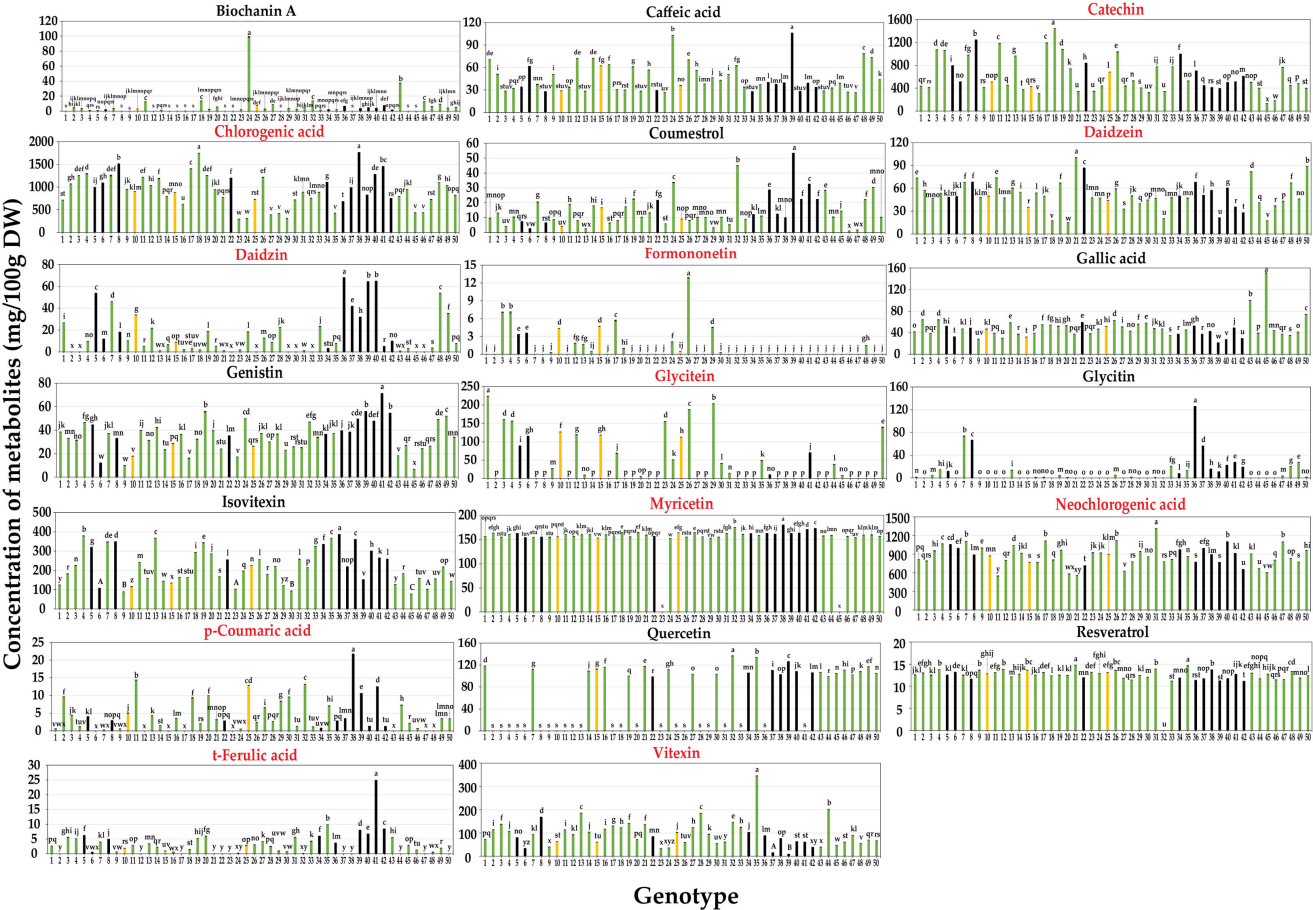
Contents of 20 secondary metabolites of mungbean sprouts on 50 genotypes. Metabolites names in red indicate the target compounds of qRT-PCR. Bar color indicates seed coat color of mungbean genotypes. Statistical analysis was conducted by Duncan test (P < 0.05).

Metabolic profiling was conducted on the 50 mungbean genotypes using a UPLC-PDA system. Of the 23 standard compounds that have been previously detected in legumes, 20 metabolites were identified in the mung bean sprout extracts. In most genotypes, catechin [(132.89 ± 0.03–1,441.46 ± 0.92 mg/100 g dry weight (DW)], chlorogenic acid (294.79 ± 0.36–1,763.24 ± 0.23 mg/100 g DW), and neochlorogenic acid (548.93 ± 1.52–1,308.58 ± 1.15 mg/100 g DW) showed the highest contents, followed by isovitexin (77.9 ± 0.05–386.73 ± 0.11 mg/100 g DW), myricetin (152.89 ± 0.03–79.82 ± 0.11 mg/100 g DW), and vitexin (9.39 ± 0.05–344.95 ± 0.15 mg/100 g DW) (**Figures 1**, **2**). In VC1973A (genotype no. 45), a reference elite mungbean cultivar, most metabolites were relatively lower than in the other genotypes, while gallic acid (99.93 ± 0.13 mg/100 g DW) was highest. In wild mungbeans (genotype no. 36–42), higher contents of isoflavones, such as daidzin, genistin, and glycitin, were detected than in the cultivated genotypes (**Supplementary Tables 3, 4**).

### 3.2 HCA and PCA based on the contents of secondary metabolites

Based on the contents of the 20 secondary metabolites, the 50 mungbean genotypes were clustered using HCA and PCA (**Figure 1**). Five clusters were grouped using HCA, and the genotypes in each cluster were consistently grouped using the PCA. Neochlorogenic acid, chlorogenic acid, catechin, isovitexin, and myricetin affected clustering the most in the order of the HCA (**Figure 1A**). The PCA revealed two principal components (PCs), with PC1 and PC2 accounting for 69.66% and 15.04% of the variance, respectively (**Figure 1B**). Chlorogenic acid, catechin, neochlorogenic acid, isovitexin, and vitexin contributed the most in that order.

### 3.3 Correlation analysis of secondary metabolites

**Figure 3:**
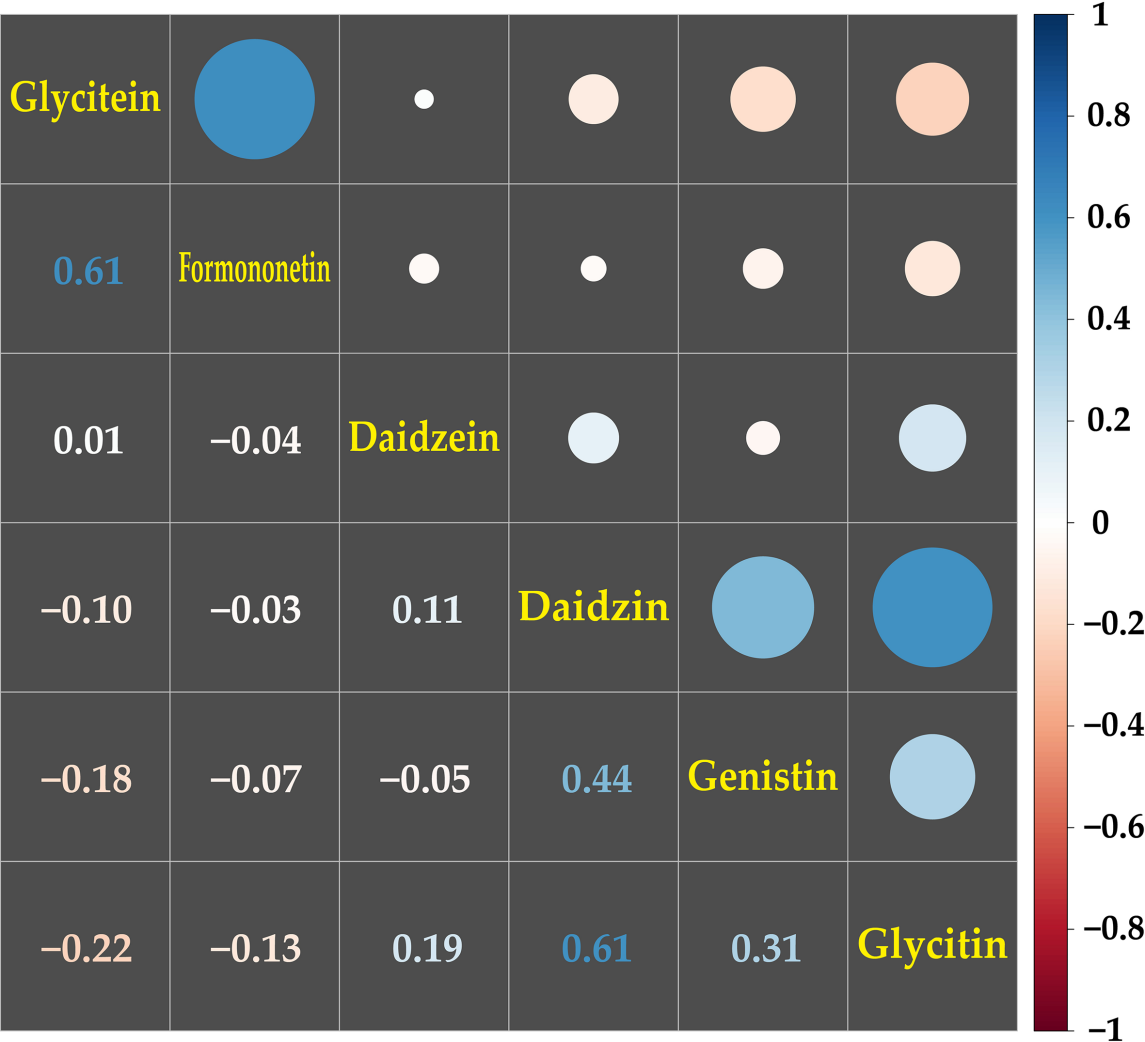
Correlation analysis among the contents of isoflavonoids in mungbean sprouts. The correlations among isoflavonoids were presented by pearson correlation coefficient and color scale from red to blue. Red indicates negative correlation, and blue indicates positive correlation between the secondary metabolites.

A correlation analysis was conducted on the contents of the 20 metabolites among the 50 genotypes. The most significant correlations were detected among the isoflavonoid groups, including daidzein, daidzin, formononetin, genistin, glycitein, and glycitin (**Figure 3**). The glycitein and formononetin contents, and the daidzin, and glycitin contents, which are synthesized by the same enzymes, isoflavonoid synthase (IFS) and isoflavone 7-O glucosyltransferase (IF7GT), respectively, in the biosynthesis pathway, showed the highest positive correlation of 0.61. The next highest positive correlations were between diadzin and genistin (correlation coefficient = 0.44) and genistin and glycitin (0.31), with IF7GT involved in the biosynthesis. Glycitein, which a precursor of glycitin, had a negative correlation of -0.22 with glycitin (**Figure 3**; **Supplementary Figure 2**).

### 3.4 Genetic factors correlated with the secondary metabolite contents

**Figure 4:**
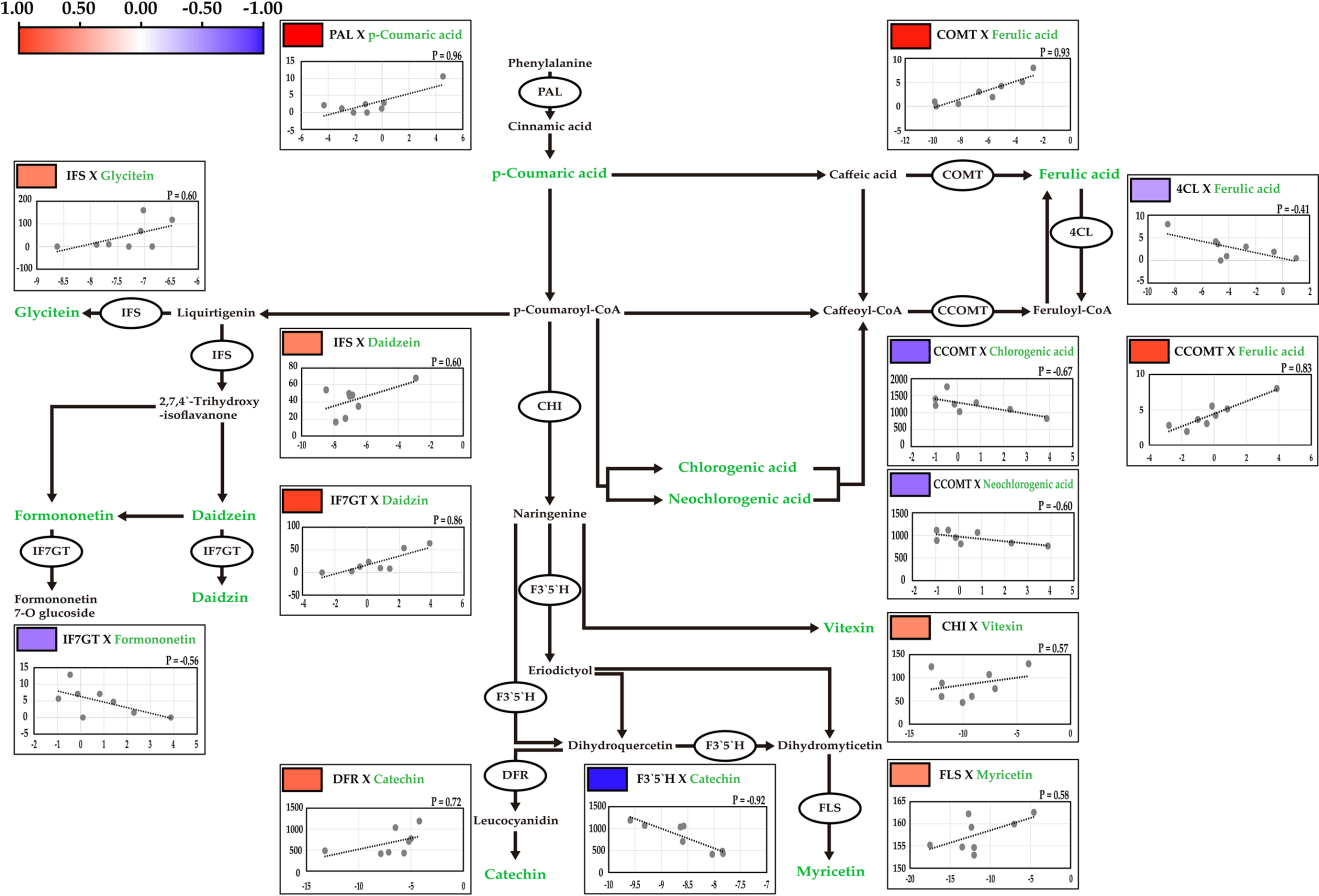
Correlation analysis between the contents of secondary metabolites and the expression levels of key genetic factors. The correlations were presented by pearson correlation with color scale from blue to red. Red and blue indicates positive and negative correlations, respectively. R is indicates pearson correlation coefficient between the metabolites and gene expression. Target metabolites are indicating green.

The candidate key genes involved in these pathways were identified using the Kyoto Encyclopedia of Genes and Genomes (KEGG) database (http://www.genome.ad.jp/kegg/). In total, the nucleotide sequences of 85 genes in model species such as *Arabidopsis thaliana* and soybean were used to identify 10 orthologous genes in mungbeans (Ha et al., 2021) (**Figure 4**).

Phenylalanine ammonia-lyase (PAL) is involved in the biosynthesis of p-coumaric acid from phenylalanine (Cochrane et al., 2004). Caffeate O-methyltransferase (COMT) catalyzes the conversion of caffeic acid into ferulic acid (Ebel et al., 1974). 4-coumarate-CoA ligase (4CL) catalyzes the conversion of ferulic acid to feruloyl-CoA (Schneider et al., 2005). Caffeoyl-CoA O-methyltransferase (CCOMT) is involved in ferulic acid biosynthesis from Caffeoyl-CoA (Kühnl et al., 1989). Isoflavone synthase (IFS) is involved in the biosynthesis of daidzein, and formononetin, and in the pathway from liquirtigenin to glycitein (Hashim et al., 1990). Isoflavone 7-O-glucosyltransferase (IF7GT) catalyzes daidzin biosynthesis (Noguchi et al., 2007). Chalcone isomerase catalyzes naringenin biosynthesis, the precursor of vitexin (Shirley et al., 1992). Flavanoid 3’, 5’-hydroxylase (F3’5’H) catalyzes the pathway from dihydroquercetin to dihydromyricetin (Shimada et al., 1999). Dihydroflavonol 4-reductase (DFR) is catalyzes in the biosynthesis of leucocyanidin, the precursor of catechin (Fischer et al., 2003). Flavonol synthase (FLS) catalyzes the conversion of dihydromyricetin to myricetin (Wellmann et al., 2002).

The relative expression levels of the candidate genes were measured in 12 genotypes, and significant variations were detected in the contents of major secondary metabolites (**Table 2**; **Supplementary Table 5**). The expression levels of the 10 candidate genes were significantly correlated with the contents of related secondary metabolites, as measured by UPLC (**Figure 4**). The highest positive correlation was between *PAL* expression and p-coumaric acid contents, with a correlation coefficient of 0.96, followed by *COMT* and ferulic acid (0.93), *IF7GT* and daidzin (0.86), and *CCOMT* and ferulic acid (0.83). The highest negative correlation was between *F3`5`H* expression and the catechin contents (-0.92). Both chlorogenic acid and neochlorogenic acid, which are isomers, negatively correlated with *CCOMT* expression, with correlation coefficients of -0.67 and -0.60, respectively.

**Table 2:** The contents of secondary metabolites in the mungbean genotypes with high variations.

Genotype No.	Catechin	Chlorogenic acid	Daidzein	Daidzin	Formononetin	Glycitein	Myricetin	Neochlorogenic acid	p-Coumaric acid	t-Ferulic acid	Vitexin
**3**	1072.7 ± 0.43^b^	1252.73 ± 0.9^cd^	46.58 ± 0.02^f^	N.D.	7.1 ± 0.04^b^	160.51 ± 0.07^b^	154.77 ± 0.02^e^	951.78 ± 2.7^c^	4.43 ± 0.03^c^	5.53 ± 0.03^b^	137.23 ± 0.31^a^
**4**	1058.66 ± 2.73^bc^	1292.84 ± 3.85^c^	53.72 ± 0.09^d^	9.78 ± 0.02^h^	7.07 ± 0.11^b^	156.2 ± 0.08^b^	159.97 ± 0.03^c^	1064.24 ± 0.63^b^	1.17 ± 0.01^g^	5.11 ± 0.01^c^	107.39 ± 0.33^c^
**15**	426.41 ± 0.26^gh^	875.89 ± 0.22^f^	35.29 ± 0.13^g^	8.62 ± 0.02^h^	4.72 ± 0.02^d^	118.32 ± 0.03^d^	152.95 ± 0.02^f^	765.52 ± 0.32^f^	N.D.	0.96 ± 0.01^h^	60.15 ± 0.03^g^
**17**	1192.24 ± 2.5^a^	1404.07 ± 4.69^b^	49.73 ± 0.12^e^	2.79 ± 0.04^i^	5.68 ± 0.07^c^	68.39 ± 0.38^e^	155.24 ± 0.03^e^	1113.38 ± 0.88^a^	N.D.	N.D.	130.31 ± 0.01^b^
**26**	1035.63 ± 1.72^c^	1215.06 ± 1.86^d^	59.33 ± 0.07^c^	12.77 ± 0.03^g^	12.89 ± 0.04^a^	187.06 ± 0.43^a^	154.66 ± 0.03^e^	1117.76 ± 0.11^a^	2.44 ± 0.02^ef^	3.04 ± 0.01^f^	59.73 ± 0.12^g^
**33**	774.73 ± 1.6^d^	883.91 ± 3.49^f^	47.75 ± 0.09^f^	23.39 ± 0.08^f^	N.D.	N.D.	160.18 ± 0.07^c^	814.42 ± 1.57^e^	1.14 ± 0.01^g^	4.21 ± 0.04^d^	124.01 ± 0.56^b^
**36**	705.13 ± 0.31^e^	682.24 ± 0.9^g^	68 ± 0.04^b^	67.97 ± 0.13^a^	N.D.	10.29 ± 0.02^f^	162.62 ± 0.02^b^	772.66 ± 0.07^f^	2.79 ± 0.01^e^	3.62 ± 0.01^e^	88.49 ± 0.19^d^
**38**	414.59 ± 0.07^hi^	1763.24 ± 0.23^a^	57.4 ± 0.03^c^	31.94 ± 0.05^e^	N.D.	N.D.	179.82 ± 0.11^a^	889.73 ± 0.26^d^	21.72 ± 0.05^a^	N.D.	76.57 ± 0.25^e^
**39**	397.8 ± 0.26^i^	827.67 ± 0.33^f^	21.57 ± 0.01^h^	64.43 ± 0.07^b^	N.D.	N.D.	162.26 ± 0.05^b^	766.23 ± 0.39^f^	10.6 ± 0.01^b^	8.01 ± 0.02^a^	9.39 ± 0.05^i^
**45**	132.89 ± 0.03^j^	439.14 ± 0.41^h^	17.44 ± 0.03^i^	N.D.	N.D.	8.6 ± 0.04^f^	N.D.	602.15 ± 0.28^g^	2.15 ± 0.01^f^	2.78 ± 0.02^f^	46.87 ± 0.05^h^
**48**	447.44 ± 0.17^g^	1097.48 ± 1.43^e^	67.08 ± 0^f^	53.93 ± 0.06^c^	1.48 ± 0.01^e^	N.D.	158.71 ± 0.02^d^	832.52 ± 0.55^f^	N.D.	0.51 ± 0.01^i^	55.03 ± 0.1^f^
**49**	484.83 ± 0.41^f^	1032.23 ± 1.49^f^	46 ± 0.01^a^	35.13 ± 0.07^d^	N.D.	N.D.	159.25 ± 0.07^cd^	771.86 ± 0.25^c^	3.49 ± 0.01^d^	1.92 ± 0.01^g^	69.62 ± 0.02^f^

N.D., not detected. (mg/100g DW).

## 4 Discussion

Legume crops have high nutritional and health benefits and can produce high yields over relatively short cultivation periods, which are important traits for food crops (Iqbal et al., 2006; Rebello et al., 2014). Legume crops contain high amounts of secondary metabolites, including flavonoids and isoflavones, which can positively and negatively affect food quality. On the positive side, secondary metabolites have antioxidant, anti-inflammatory, and anti-cancer effects in humans (Ali Reza et al., 2021). However, some secondary metabolites can lower the food quality, taste bitter, decrease legume storage capacity, and discolor food (Oz et al., 2017). In this study, 20 secondary metabolites were profiled in 50 mungbean genotypes using UPLC to provide useful information for improving the food quality of mungbean sprouts.

Neochlorogenic acid was the most abundant component in most genotypes, followed by chlorogenic acid and catechin identified in the HCA (**Figure 1A**), whereas chlorogenic acid, catechin, and neochlorogenic acid were the major components in the PCA (**Figure 1B**). Although neochlorogenic acid was the most abundant secondary metabolite, it had less of an effect on clustering in the PCA because catechin and chlorogenic acid had higher variances among the genotypes (**Figure 2**). Among the 20 functional substances, the chlorogenic acid and catechin contents showed the greatest variation, suggesting that these two compounds could be the first targets of genetic engineering or molecular breeding to improve the nutritional value of mungbean sprouts.

The mungbean reference genotype VC1973A (genotype 45) mungbean contained the highest amount of gallic acid (**Figure 2**). Gallic acid is known to have effects of antioxidant, anti-inflammatory and neuroprotective capacity, that are beneficial for human health (Kroes et al., 1992; Lu et al., 2006). However, VC1973A had relatively lower contents of most compounds than the other genotypes. Commercial strawberry (*Fragaria* × *ananassa*) cultivars have lower anthocyanin contents and diversity than non-commercial cultivars and wild types, which is one of the problems to be addressed in breeding programs (Dzhanfezova et al., 2020). Wild mungbeans (genotype no. 36–42) had relatively higher isoflavone contents, including daidzein, genistin, and glycitin, than other genotypes, including VC1973A (**Figure 2**). Isoflavones have physiological functions, such as phytoestrogens that have beneficial effects on bone health and reduce menopausal symptoms and the risk of breast cancer (Chen and Chen, 2021). Thus, the results of this study suggest that wild mungbeans can be a useful resource for producing functional substances that promote the nutritional value of mungbean sprouts.

Among the secondary metabolites identified in the mungbean sprouts, isoflavones, including daidzein, formononetin, genistin, glycitein, and glycitin, showed relatively high positive correlations (**Figure 3**). The expression levels of transcripts encoding key enzymes in the biosynthetic pathways of isoflavones were measured using qRT-PCR (**Figure 4**). Positive correlations were identified between secondary metabolite contents and the expression of genes that catalyze the biosynthetic pathways of secondary metabolites, such as *IFS* and daidzein, and *IF7GT* and daidzin. Soybean *IFS* has been shown to catalyze the biosynthesis of daidzein and genistein, which are the precursors of daidzin and genistin in rapeseed (Li et al., 2011). In contrast, negative correlations were detected between the formononetin content and *IF7GT* expression levels, where formononetin is catalyzed by formononetin 7-O glucoside by *IF7GT*. In soybeans, the aglycosylation of isoflavones decreased as the expression of *IF7GT* decreased (Gupta et al., 2017). A negative correlation was reported between formononetin contents and expression level of *IF7GT* because *IF7GT* is associated with the aglycosylation of formononetin (De Vega et al., 2015). The catechin contents and expression levels of *F3’5’H*, which catalyzes the other side of the biosynthetic pathway of dihydroquercetin, a precursor of catechin, also had a negative correlation (**Figure 4**). *F3’5’H* had been reported to catalyze the biosynthesis of dihydromyricetin from dihydroquercetin in grape (*Vitis vinifera*) and tea plant (*Camellia sinensis*) (Jeong et al., 2006; Zhou et al., 2016). Within the pathway, the biosynthesis of delphinidin-based anthocyanins and cyanidin-based anthocyanins increased and decreased, respectively, as the expression level of *F3’5’H* increased. When dihydroquercetin, a precursor of leucocyanidin, was synthesized into dihydromyricetin by *F3’5’H*, the contents of leucocyanidin decreased. Thus, in the current study, when the expression level of *F3’5’H* increased, the leucocyanidin contents decreased, which is a precursor of catechin, resulting in a negative correlation between the catechin contents and expression of F3’5’H. In summary, significant correlations were identified between the contents of secondary metabolites and the expression levels of the genes catalyzing the biosynthetic pathways of these metabolites, indicating that the secondary metabolite contents are regulated at the level of the transcription of key genes in their pathways in mungbeans. These results suggest that functional substance content can be regulated by manipulating target genes for molecular breeding or genetic engineering.

In this study, metabolic profiling and transcriptome analysis were conducted using UPLC and qRT-PCR, respectively, on 50 mungbean genotypes of different origins. The key genes involved in the biosynthesis of secondary metabolites with beneficial effects on human health were identified in the mungbean sprouts. We found that wild mungbeans contained significantly higher levels of isoflavones, such as daidzin, genistin, and glycitin, than cultivated mungbeans. The chlorogenic acid and catechin contents showed the highest variance among the genotypes. A strong positive correlation was detected among the isoflavone groups in which the mainstream biosynthetic pathways were regulated by the same key enzymes (**Figure 4**; **Supplementary Figure 2**). The results of this study will help enable improving the nutritional value of mungbean sprouts containing functional substances for desired purposes while reducing secondary metabolites that negatively impact food quality.

## Data availability statement

The original contributions presented in the study are included in the article/**Supplementary Material**. Further inquiries can be directed to the corresponding author.

## Author contributions

BK designed the experiments and wrote the manuscript. BK and IL collected the material data and conducted experiments. BK performed the qualifying and quantifying of the metabolites and analyzed the transcriptomic results. IL contributed the transcriptomic results analyse. JH supervised and revised the manuscript. All authors contributed to the article and approved the submitted version.
