# Supplementary material for: Metabolic profiling and expression analysis of key genetic factors in the biosynthetic pathways of antioxidant metabolites in mungbean sprouts
Source: Front Plant Sci. 2023 Jun 16;14:1207940. doi: 10.3389/fpls.2023.1207940 (PMC10313209; doi:10.3389/fpls.2023.1207940)
Supplement: Supplementary file 1 [file DataSheet_1.docx]

Supplementary Material

**Supplementary table 1.** List of mungbean genotypes.

| **Number.** | **Accession name.** | **Origin** | **Number.** | **Accession name.** | **Origin** | **Number.** | **Accession name.** | **Origin** |
| --- | --- | --- | --- | --- | --- | --- | --- | --- |
| **1** | JP229145 | CHN | **18** | Vo1301 | CHN | **35** | Fue Nutu | IDN |
| **2** | JP229215 | CHN | **19** | Vo3484 | PAK | **36** | W147 |  |
| **3** | JP229216 | CHN | **20** | Vo5551 | IRN | **37** | W162 |  |
| **4** | JP229099 | THA | **21** | Collected Yecheon-1992 | KOR | **38** | W169 |  |
| **5** | JP229177 | IND | **22** | Damyang, Jeollanam-do-1994-3231 | KOR | **39** | W176 |  |
| **6** | JP229175 | IND | **23** | Namwon, Jeollabuk-do-1994-3237 | KOR | **40** | W190 |  |
| **7** | JP103138-1 | PAK | **24** | V03720B-G | USA | **41** | W191 |  |
| **8** | JP103138-2 | PAK | **25** | ACC11 | PHL | **42** | W192 |  |
| **9** | JP99066 | PAK | **26** | Acc. 363 | PHL | **43** | Dahyeon | KOR |
| **10** | Bohabe yellow mongo | PHL | **27** | V01471 | IDN | **44** | Samgang | KOR |
| **11** | JP229254 | IRN | **28** | ML-9 | CHN | **45** | VC1973A | TWN |
| **12** | CN900001 | THA | **29** | VC-2307A | CHN | **46** | SM1409 |  |
| **13** | Suwon No.2 | KOR | **30** | CHN-PMW-2011-4 | CHN | **47** | SM1501 |  |
| **14** | Gimje, Jeollabuk-do-1985-3835 | KOR | **31** | Arta ijo | IDN | **48** | V2984 | KOR |
| **15** | Yellowgram | UNK | **32** | Betet | IDN | **49** | IT208075 | VNM |
| **16** | Boeun, Chungcheong buk-do-1989-5499 | KOR | **33** | PB-1  (benggolo Puti) | IDN | **50** | Jeongdong Sanghoe |  |
| **17** | Imsil, Jeollabuk-do-1989-5600 | KOR | **34** | Tecer Hitam | IDN |  |  |  |


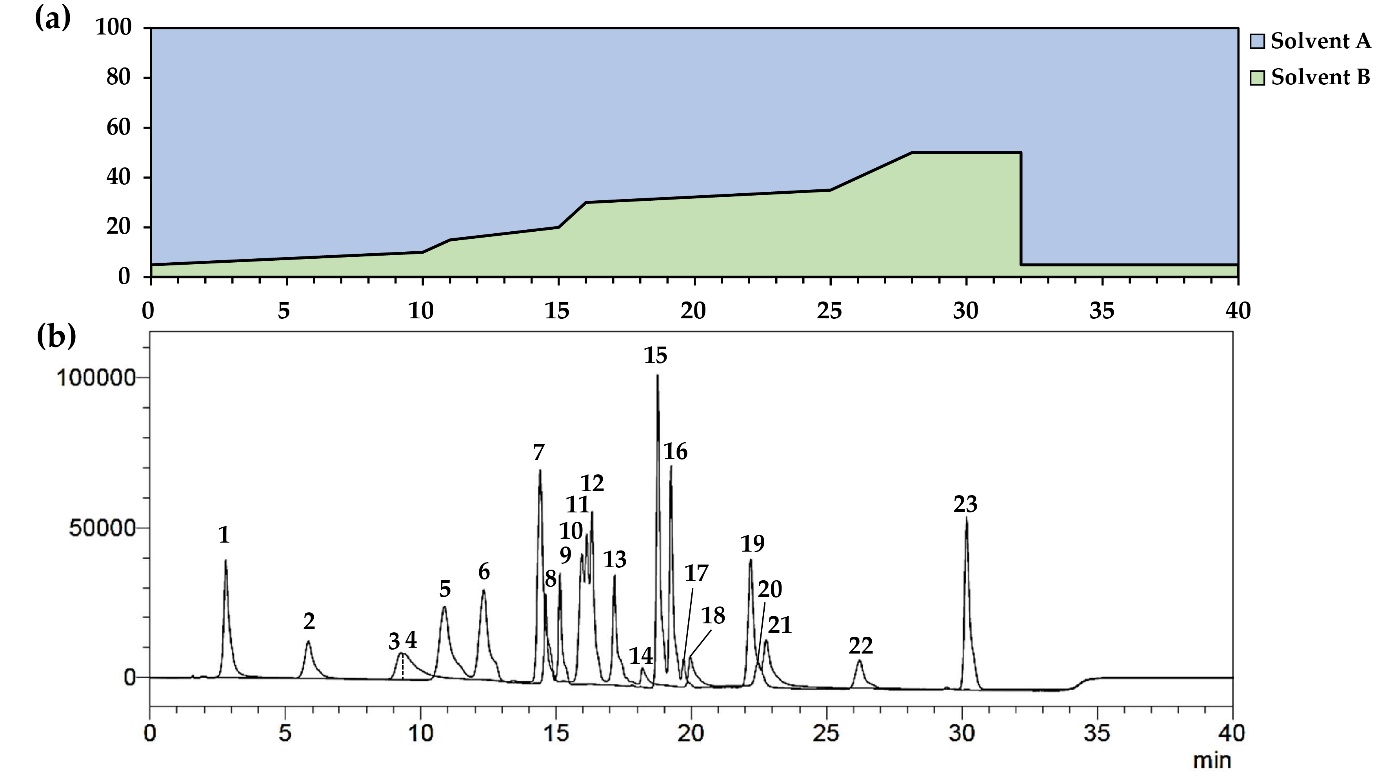


**Supplementary figure 1.** UPLC-PDA mobile page gradient condition and standard compounds peak. Solvent types were indicates by colors. Blue and green indicates solvent A (water-acetic acid 0.1%), and B (ACN), respectively.

**Supplementary table 2**. qRT-PCR primer list.

| **Gene** | **Primer** | **Primer sequences (5'-3')** |
| --- | --- | --- |
| **PAL** | Forward | GTCTCCATGGACAACACACG |
|  | Reverse | GGATTTCTGCTAGCGGTGAG |
| **F3'5'H** | Forward | TGATGCACAGGACATGGTTT |
|  | Reverse | CCTTTCCCCACTCATCAAGA |
| **IF7GT** | Forward | GAGATCGCTATTGGGTTGGA |
|  | Reverse | TCCTCTCCAGAAACCCCTCT |
| **DFR** | Forward | GTGCAAAGAGCAAGCTGTCA |
|  | Reverse | GTGTGGCCAAGTGGAAAACT |
| **COMT** | Forward | GTGGTGGTCTTGGGATCAAC |
|  | Reverse | ATATCTCCGCAAACGTGGTC |
| **CCOMT** | Forward | CATATTTGTGGATGCGGACA |
|  | Reverse | AACCGTTCCATAGGGTGTTG |
| **CHI** | Forward | AGCCACCGGAAAAACCTATT |
|  | Reverse | GTCACCGCTTTATCCTCCAA |
| **IFS** | Forward | GCAATAGGGTTGTTGGTGCT |
|  | Reverse | AAAGGTGAAGGTGTCCAACG |
| **4CL** | Forward | ACGGAAGAGGCTGTGAAAGA |
|  | Reverse | CCGATGGAGACTTGGGAATA |
| **FLS** | Forward | TGAAAGGGGTGAACCTTGAG |
|  | Reverse | TTGGAACATTCCCCACTCTC |

**Supplementary table 3.** Contents of phenylpropanoid, flavan and flavonols in 50 mungbean genotypes. (mg/100g DW)

| **Genotype No.** | **Caffeic acid** | **Catechin** | **Chlorogenic acid** | **Gallic acid** | **Myricetin** | **Neochlorogenic acid** | **p-Coumaric acid** | **Quercetin** | **Resveratrol** | **t-Ferulic acid** |
| --- | --- | --- | --- | --- | --- | --- | --- | --- | --- | --- |
| **1** | 70.65±0.05 | 436.97±0.53 | 714.03±0.82 | 41.72±0.06 | 155.82±0.05 | 810.71±0.64 | 0.56±0 | 118.2±0.04 | 12.58±0.01 | 2.61±0 |
| **2** | 51.22±0.14 | 416.16±0.32 | 1071.85±0.31 | 63.93±0.05 | 163.13±0.06 | 789.76±1.45 | 9.64±0.03 | N.D. | 13.01±0.01 | N.D. |
| **3** | 28.47±0.02 | 1072.7±0.43 | 1252.73±0.9 | 38.47±0.05 | 154.77±0.02 | 951.78±2.7 | 4.43±0.03 | N.D. | 12.53±0.01 | 5.53±0.03 |
| **4** | 32.1±0.15 | 1058.66±2.73 | 1292.84±3.85 | 63.82±0.07 | 159.97±0.03 | 1064.24±0.63 | 1.17±0.01 | N.D. | 13.8±0.01 | 5.11±0.01 |
| **5** | 33.55±0.37 | 795.63±0.34 | 992.33±3.18 | 52.19±0.06 | 162.35±0.07 | 1054.93±0.99 | 3.99±0.02 | N.D. | 12.52±0.03 | 6.22±0.01 |
| **6** | 61.62±0.11 | 513.72±0.68 | 1094.68±0.65 | 32.35±0.03 | 153.94±0.04 | 993.32±0.68 | N.D. | N.D. | 13.2±0.01 | 0.56±0.01 |
| **7** | 37.26±0.41 | 983.03±1.04 | 1254.82±10.05 | 47.03±0.14 | 154.45±0.05 | 1105.89±0.48 | 0.25±0 | 112.58±0.07 | 12.52±0 | 3.94±0.01 |
| **8** | 28.05±0.03 | 1243.16±2.57 | 1506.7±5.79 | 48.81±0.08 | 154.99±0.04 | 888.34±0.43 | 2.9±0.01 | N.D. | 11.63±0.01 | 4.91±0.02 |
| **9** | 50.94±0.05 | 414.52±0.28 | 953.49±0.43 | 28.18±0.03 | 154.61±0.03 | 997.77±0.62 | 0.6±0.01 | N.D. | 13.66±0 | 0.66±0 |
| **10** | 28.72±0.02 | 509.67±0.09 | 904.69±0.42 | 46.85±0.11 | 155.44±0.01 | 869.98±0.37 | 4.99±0.04 | N.D. | 12.83±0 | 1.87±0.01 |
| **11** | 34±0.18 | 1184.68±2.38 | 1216.18±1.26 | 39.06±0.07 | 160.03±0.1 | 548.93±1.52 | 14.38±0.04 | N.D. | 13.1±0 | 2.8±0.01 |
| **12** | 72.14±0.05 | 455.18±0.09 | 1031.88±1.47 | 29.87±0.08 | 156.38±0.05 | 800.24±0.88 | N.D. | N.D. | 13.84±0.01 | N.D. |
| **13** | 28.55±0.03 | 964.96±0.58 | 1190.75±5.25 | 58.53±0.06 | 159.47±0.05 | 1036.86±1.01 | 4.32±0.01 | N.D. | 12.12±0.01 | 3.38±0.01 |
| **14** | 72.36±0.02 | 376.3±0.26 | 792.99±0.15 | 37.1±0.02 | 159.83±0.02 | 914.31±0.28 | 1.47±0.01 | 109.15±0.02 | 12.74±0.01 | 2.27±0.01 |
| **15** | 62.2±0.01 | 426.41±0.26 | 875.89±0.22 | 32.03±0.12 | 152.95±0.02 | 765.52±0.32 | N.D. | 112.8±0.01 | 13.62±0.01 | 0.96±0.01 |
| **16** | 63.75±0 | 307.01±0.21 | 61.98±0.08 | 39.16±0.02 | 159.36±0.05 | 766.12±0.22 | 3.59±0 | 116.32±0.07 | 12.57±0 | 0.51±0 |
| **17** | 30.4±0.1 | 1192.24±2.5 | 1404.07±4.69 | 54.94±0.13 | 155.24±0.03 | 1113.38±0.88 | N.D. | N.D. | 13.13±0.02 | N.D. |
| **18** | 30±0.07 | 1441.46±0.92 | 1747.66±0.88 | 54.41±0.11 | 164.1±0.05 | 804.09±1.26 | 9.35±0.11 | N.D. | 12.41±0 | 1.5±0.02 |
| **19** | 60.88±0.03 | 1076.08±1.52 | 1252.84±2.67 | 51.93±0.08 | 155.38±0.02 | 964.51±0.4 | 2.04±0.01 | 99.97±0.02 | 12.52±0.01 | 5.16±0.01 |
| **20** | 28.6±0.01 | 741.1±0.57 | 948.17±0.51 | 52.9±0.04 | 163.84±0.1 | 581.21±2.27 | 9.94±0 | N.D. | 12.5±0.01 | 5.93±0.05 |
| **21** | 56.77±0.03 | 344.32±0.28 | 773.06±0.52 | 37.44±0.05 | 158.98±0.01 | 558.5±0.22 | 3.15±0.02 | 117.28±0.02 | 14.75±0 | N.D. |
| **22** | 29.54±0.03 | 839.62±0.62 | 1201.04±1.33 | 59.41±0.05 | 156.18±0.01 | 715.13±1.24 | 2.84±0.01 | 98.73±0.03 | 11.95±0 | N.D. |
| **23** | 26.85±0 | 347.89±0.78 | 294.79±0.36 | 37.97±0.13 | N.D. | 921.44±2.13 | N.D. | N.D. | 13.14±0 | N.D. |
| **24** | 102.84±0.01 | 442.48±0.19 | 314.6±0.29 | 46.75±0.02 | 151.61±0.01 | 918.16±0.21 | 0.52±0 | 111.87±0.03 | 12.88±0 | 0.21±0.01 |
| **25** | 36.06±0.01 | 683.88±0.71 | 731.47±0.52 | 51.78±0.06 | 163.68±0.1 | 896.65±0.28 | 12.89±0.03 | N.D. | 13.07±0 | 2.87±0.02 |
| **26** | 70.32±0.06 | 1035.63±1.72 | 1215.06±1.86 | 62.85±0.03 | 154.66±0.03 | 1117.76±0.11 | 2.44±0.02 | N.D. | 13.61±0.02 | 3.04±0.01 |
| **27** | 56.06±0.02 | 439.01±0.25 | 388.81±0.06 | 50.6±0.1 | 164.19±0.09 | 625.82±0.34 | 6.58±0.01 | 103.42±0.03 | 11.85±0 | 4.11±0.02 |
| **28** | 37.99±0.03 | 529.96±0.49 | 422.11±0.69 | 43±0.01 | 155.33±0 | 774.18±0.35 | 2.63±0.02 | N.D. | 11.44±0 | 2.46±0.02 |
| **29** | 46.29±0.03 | 407.55±0.91 | 314.67±0.47 | 55.86±0.1 | 152.89±0.03 | 940.97±1.59 | 8.42±0.03 | N.D. | 12.48±0 | 0.94±0.02 |
| **30** | 42.97±0.04 | 322.29±0.45 | 717.8±0.25 | 57.92±0.14 | 154.81±0.08 | 854.66±0.4 | 9.62±0.01 | 103.34±0.05 | 12.09±0 | 0.75±0 |
| **31** | 50.7±0.02 | 774.22±0.95 | 888.39±1.07 | 47.5±0.13 | 162.54±0.04 | 1308.58±1.15 | 1.27±0.02 | N.D. | 13.89±0 | 5.56±0.01 |
| **32** | 62.43±0.03 | 344.35±0.13 | 754.94±0.42 | 46.9±0.04 | 174.75±0.05 | 775.34±0.32 | 13.2±0.01 | 136.81±0.07 | N.D. | 0.14±0 |
| **33** | 33.92±0.05 | 774.73±1.6 | 883.91±3.49 | 34.72±0.08 | 160.18±0.07 | 814.42±1.57 | 1.14±0.01 | N.D. | 11.19±0 | 4.21±0.04 |
| **34** | 27.92±0.01 | 997.15±1.21 | 1106.33±3.01 | 37.11±0.05 | 162.07±0.04 | 969±2.18 | 0.8±0 | 105.55±0.02 | 11.89±0.01 | 5.98±0.02 |
| **35** | 37.12±0.03 | 527.45±0.71 | 427.02±0.29 | 45.24±0.06 | 158.04±0.03 | 858.52±0.57 | 7.06±0 | 133.68±0.06 | 14.64±0.01 | 9.8±0.02 |
| **36** | 40.17±0.01 | 705.13±0.31 | 682.24±0.9 | 52.73±0.08 | 162.62±0.02 | 772.66±0.07 | 2.79±0.01 | N.D. | 11.34±0.01 | 3.62±0.01 |
| **37** | 37.56±0.04 | 447.93±0.3 | 990.76±0.7 | 37.04±0.08 | 161.08±0.03 | 990.13±0.83 | 3.5±0.01 | 110.45±0.03 | 11.73±0.01 | N.D. |
| **38** | 39.44±0.04 | 414.59±0.07 | 1763.24±0.23 | 42.35±0.05 | 179.82±0.11 | 889.73±0.26 | 21.72±0.05 | 102.79±0.03 | 13.74±0.01 | N.D. |
| **39** | 106.05±0 | 397.8±0.26 | 827.67±0.33 | 21.25±0.04 | 162.26±0.05 | 766.23±0.39 | 10.6±0.01 | 126.19±0.08 | 11.23±0 | 8.01±0.02 |
| **40** | 28.25±0 | 500.15±1.36 | 1279.94±0.25 | 27.63±0.01 | 163.05±0.06 | 1099.04±0.36 | 1.33±0.01 | 108.31±0.06 | 11.72±0 | 6.69±0.03 |
| **41** | 39.22±0.02 | 513.63±0.06 | 1454.56±0.66 | 48.86±0.11 | 170.69±0.01 | 910.75±0.54 | 12.5±0 | N.D. | 12.72±0.02 | 24.93±0.01 |
| **42** | 33.79±0 | 611.38±0.09 | 749.58±0.86 | 29.4±0.02 | 172.58±0.06 | 654.24±0.23 | 1.27±0.01 | 106.01±0.07 | 11.14±0 | 8.43±0.02 |
| **43** | 28.98±0.02 | 504.12±0.55 | 794.82±0.62 | 99.93±0.13 | 157.11±0.06 | 902.26±0.54 | N.D. | 106.61±0.03 | 12.96±0 | 5.49±0.01 |
| **44** | 32.68±0.01 | 403.67±0.52 | 944.65±0.22 | 39.21±0.08 | 158.36±0.01 | 667.3±0.12 | 7.27±0.01 | 98.85±0.02 | 11.68±0 | N.D. |
| **45** | 39.1±0.03 | 132.89±0.03 | 439.14±0.41 | 151.16±0.12 | N.D. | 602.15±0.28 | 2.15±0.01 | 104.92±0.08 | 12.76±0.01 | 2.78±0.02 |
| **46** | 27.46±0.01 | 183.65±0.21 | 438.07±0.05 | 44.06±0.05 | 156.15±0.03 | 804.88±0.05 | 0.72±0 | 110.98±0.04 | 11.44±0 | 1.29±0.01 |
| **47** | 26.29±0.01 | 763.96±0.48 | 727.57±0.58 | 37.2±0.05 | 153.66±0.04 | 1097.25±0.27 | N.D. | 101.97±0.04 | 11.55±0 | N.D. |
| **48** | 78.51±0.06 | 447.44±0.17 | 1097.48±1.43 | 34.96±0.11 | 158.71±0.02 | 832.52±0.55 | N.D. | 107.95±0.04 | 13.38±0.01 | 0.51±0.01 |
| **49** | 73.17±0.07 | 484.83±0.41 | 1032.23±1.49 | 41.2±0.11 | 159.25±0.07 | 771.86±0.25 | 3.49±0.01 | 117.02±0.04 | 11.87±0.01 | 1.92±0.01 |
| **50** | 43.6±0.01 | 400.57±0.97 | 819.87±0.16 | 73.98±0.2 | 156.59±0.01 | 961.35±0.16 | 3.43±0.01 | 104.87±0.02 | 12.4±0 | N.D. |

N.D.: not detected.

**Supplementary table 4.** Contents of Isoflavonoids and flavones in 50 mungbean genotypes. (mg/100g DW)

| **Genotype No.** | **BiochaninA** | **Coumestrol** | **Daidzein** | **Daidzin** | **Formononetin** | **Genistin** | **Glycitein** | **Glycitin** | **Isovitexin** | **Vitexin** |
| --- | --- | --- | --- | --- | --- | --- | --- | --- | --- | --- |
| **1** | N.D. | 9.66±0.03 | 73.6±0.02 | 26.6±0.05 | N.D. | 38.49±0.06 | 223.43±0.37 | 1.83±0 | 124.87±0.15 | 72.96±0.11 |
| **2** | 4.46±0.02 | 13.1±0.01 | 57.39±0.02 | N.D. | N.D. | 33.11±0.06 | N.D. | N.D. | 182.28±0.12 | 114.62±0.32 |
| **3** | 3.54±0.03 | 4.15±0.03 | 46.58±0.02 | N.D. | 7.1±0.04 | 31.45±0.19 | 160.51±0.07 | 4.2±0.02 | 225.51±0.19 | 137.23±0.31 |
| **4** | 1.23±0.01 | 10.45±0.03 | 53.72±0.09 | 9.78±0.02 | 7.07±0.11 | 46.3±0.09 | 156.2±0.08 | 14.37±0.07 | 379.22±0.41 | 107.39±0.33 |
| **5** | 0.86±0.01 | 7.3±0.07 | 48.39±0.23 | 53.66±0.14 | 3.34±0 | 44.48±0.11 | 88.08±1.08 | 12.05±0.02 | 320.24±0.38 | 80.54±0.23 |
| **6** | 1.95±0.01 | 2.74±0 | 49.24±0.04 | 11.93±0.02 | 3.55±0.02 | 12.16±0.05 | 114.71±0.52 | N.D. | 107.66±0.1 | 33.88±0.13 |
| **7** | 3.63±0.01 | 20.51±0.04 | 67.75±0.02 | 46.14±0.01 | N.D. | 37.18±0.13 | N.D. | 73.66±0.26 | 347.29±0.47 | 92.8±0 |
| **8** | N.D. | 6.66±0.02 | 68.13±0.18 | 18.08±0.05 | N.D. | 32.96±0.12 | N.D. | 66.29±0.22 | 349.12±0.43 | 168.46±0.46 |
| **9** | N.D. | 8.69±0.02 | 48.3±0.01 | 10.09±0.05 | 0.31±0 | 9.97±0.08 | 27.51±0.21 | N.D. | 89.57±0.03 | 40.43±0.02 |
| **10** | 2.93±0.01 | 4.2±0.06 | 49.78±0.01 | 33.89±0.04 | 4.39±0.01 | 17.87±0.05 | 126.56±0.14 | N.D. | 116.3±0.03 | 63.44±0.07 |
| **11** | 12.47±0.04 | 18.95±0.06 | 73.77±0.02 | 5.18±0.02 | N.D. | 39.77±0.08 | N.D. | N.D. | 241.92±0.07 | 113.1±0.08 |
| **12** | N.D. | 8.07±0.02 | 47.4±0.01 | 21.79±0.06 | 1.88±0 | 31.24±0.06 | 119.04±0.23 | N.D. | 159.2±0.1 | 92.57±0.15 |
| **13** | 1.39±0.01 | 2.57±0 | 60.24±0.06 | 1.08±0.01 | 1.68±0 | 42.17±0.13 | 9.37±0.03 | 13.87±0.03 | 366.8±0.22 | 185.89±0.25 |
| **14** | N.D. | 18.03±0.05 | 54.87±0.03 | 6.75±0.03 | 0.46±0 | 23.28±0.08 | N.D. | N.D. | 145.42±0.04 | 104.47±0.02 |
| **15** | N.D. | 16.66±0.03 | 35.29±0.13 | 8.62±0.02 | 4.72±0.02 | 28.69±0.02 | 118.32±0.03 | N.D. | 134.99±0.04 | 60.15±0.03 |
| **16** | 0.16±0 | 6.4±0.02 | 53.89±0.03 | 2.16±0.01 | N.D. | 36.54±0.08 | N.D. | 1.36±0.02 | 163.92±0.01 | 116.96±0.04 |
| **17** | N.D. | 8.07±0.06 | 49.73±0.12 | 2.79±0.04 | 5.68±0.07 | 16.2±0.13 | 68.39±0.38 | 1.46±0.02 | 162.72±0.39 | 130.31±0.01 |
| **18** | 13.76±0.02 | 17.35±0.03 | 17.42±0.03 | 1.98±0.01 | 0.97±0.01 | 32.41±0.08 | N.D. | N.D. | 293.27±0.17 | 122.81±0.17 |
| **19** | 2.22±0.05 | 22.51±0.18 | 67.01±0.17 | 18.64±0.07 | N.D. | 55.69±0.24 | N.D. | 3.9±0.01 | 342.31±1.24 | 141.06±0.11 |
| **20** | 5.7±0.01 | 10.39±0.05 | 15.14±0.04 | 5.23±0.01 | N.D. | 39.49±0.02 | 5.36±0 | 0.87±0 | 285.28±0.11 | 71.88±0.1 |
| **21** | N.D. | 13.19±0.04 | 100.65±0.03 | 1±0 | N.D. | 24.07±0.08 | N.D. | N.D. | 167.84±0.11 | 137.09±0.15 |
| **22** | N.D. | 21.69±0.09 | 86.81±0.05 | N.D. | N.D. | 35.25±0.13 | N.D. | N.D. | 255.36±0.1 | 84.53±0.12 |
| **23** | 2.16±0.01 | 5.85±0.03 | 47.61±0.04 | 1.89±0.02 | N.D. | 17.17±0.04 | 155.06±0.04 | N.D. | 102.68±0.07 | 32.67±0.11 |
| **24** | 99.01±0.5 | 33.59±0.14 | 47.23±0.03 | 18.26±0.03 | 2.09±0.01 | 49.74±0.01 | 51.07±0.13 | N.D. | 196.96±0.01 | 36.15±0.08 |
| **25** | 7.63±0.01 | 9.06±0.01 | 44.14±0.02 | N.D. | 0.5±0.01 | 26.33±0.06 | 112.3±0.11 | N.D. | 225.99±0.06 | 102.84±0.13 |
| **26** | 2.85±0.01 | 8.19±0.02 | 59.33±0.07 | 12.77±0.03 | 12.89±0.04 | 37.13±0.15 | 187.06±0.43 | 4.99±0.02 | 256.95±0.25 | 59.73±0.12 |
| **27** | 8.7±0 | 10.11±0.03 | 32.91±0.03 | 8.72±0.02 | N.D. | 30.11±0.02 | N.D. | N.D. | 180.43±0.03 | 123.79±0.13 |
| **28** | N.D. | 10.18±0.05 | 50.15±0.03 | 22.51±0.07 | N.D. | 36.6±0.12 | N.D. | 1.59±0.03 | 221.32±0.14 | 185.13±0.07 |
| **29** | 3.7±0.01 | 3.24±0.01 | 39.95±0.02 | N.D. | 4.5±0.03 | 23±0.01 | 203.31±0.14 | N.D. | 121.89±0.04 | 94.67±0.13 |
| **30** | 2.68±0.01 | 10.35±0.03 | 44.87±0.04 | N.D. | 0.32±0 | 25.87±0.03 | 40.58±0.12 | N.D. | 93.65±0.04 | 55.36±0.15 |
| **31** | 4.42±0.04 | 5.31±0.03 | 46.59±0.08 | 1.27±0.02 | N.D. | 25.11±0.21 | 13.85±0.05 | 0.82±0 | 258.41±0.17 | 61.24±0.1 |
| **32** | 13.6±0.02 | 45.04±0.11 | 20.22±0.03 | N.D. | N.D. | 46.9±0.02 | N.D. | N.D. | 214.2±0.06 | 146.22±0.17 |
| **33** | 1.41±0.02 | 8.92±0.03 | 47.75±0.09 | 23.39±0.08 | N.D. | 33.78±0.13 | N.D. | 20.89±0.07 | 324.56±0.02 | 124.01±0.56 |
| **34** | 2.13±0.01 | 12.13±0.04 | 49.8±0.11 | 3.2±0.01 | N.D. | 36.55±0.06 | N.D. | 7.88±0.01 | 335.55±0.06 | 102.79±0.36 |
| **35** | 2.06±0.02 | 11.15±0.05 | 47.09±0.04 | 7.77±0.05 | N.D. | 37.21±0.01 | 48.96±0.11 | 13.15±0.02 | 366.55±0.11 | 344.95±0.15 |
| **36** | 6.71±0 | 28.61±0.08 | 68±0.04 | 67.97±0.13 | N.D. | 39.58±0.02 | 10.29±0.02 | 125.86±0.09 | 386.73±0.11 | 88.49±0.19 |
| **37** | N.D. | 12.38±0.02 | 50.91±0 | 41.95±0.06 | N.D. | 38.51±0.08 | N.D. | 56.38±0.03 | 219.43±0.07 | 15.92±0.02 |
| **38** | 3.73±0.01 | 9.95±0.07 | 57.4±0.03 | 31.94±0.05 | N.D. | 49.46±0.04 | N.D. | 15.76±0.01 | 361±0.13 | 76.57±0.25 |
| **39** | 4.68±0.01 | 53.34±0.03 | 21.57±0.01 | 64.43±0.07 | N.D. | 56.02±0.05 | N.D. | 10.91±0.05 | 152.69±0.05 | 9.39±0.05 |
| **40** | 3.84±0.01 | 22.38±0.03 | 60.45±0.02 | 64.85±0.02 | N.D. | 47.7±0.13 | N.D. | 22.29±0.04 | 301.1±0 | 63.27±0.12 |
| **41** | 7.64±0 | 32.6±0.02 | 35.61±0.02 | 5.25±0.01 | N.D. | 71.18±0.04 | 70.3±0.03 | 27.85±0.07 | 265.3±0.1 | 61.91±0.11 |
| **42** | 1.76±0.02 | 22.36±0.02 | 28.05±0.01 | 9.83±0.02 | N.D. | 54.36±0.03 | N.D. | 19.4±0.04 | 258.54±0.12 | 38.59±0.05 |
| **43** | 37.37±0.14 | 28.28±0.08 | 81.75±0.08 | 0.88±0 | N.D. | 18.27±0.04 | N.D. | N.D. | 126.79±0.09 | 39.88±0.1 |
| **44** | N.D. | 10.42±0.05 | 41.07±0.02 | 3.27±0.02 | N.D. | 27.13±0.08 | 38.28±0.29 | N.D. | 183.45±0.08 | 201.93±0.15 |
| **45** | N.D. | 14.46±0.01 | 17.44±0.03 | N.D. | N.D. | 6.75±0.01 | 8.6±0.04 | N.D. | 77.9±0.05 | 46.87±0.05 |
| **46** | 12.85±0.01 | 1.11±0.01 | 36.97±0.02 | N.D. | N.D. | 24.49±0.06 | N.D. | N.D. | 158.89±0.11 | 61.59±0.06 |
| **47** | 6.45±0.01 | 1.71±0.03 | 43.18±0.01 | 3.69±0.02 | N.D. | 26.36±0.02 | N.D. | 3.87±0.02 | 103.28±0.06 | 90.15±0.1 |
| **48** | 9.44±0.03 | 22.09±0.07 | 67.08±0 | 53.93±0.06 | 1.48±0.01 | 49.09±0.1 | N.D. | 20.32±0.02 | 156.45±0.04 | 55.03±0.1 |
| **49** | 4.14±0.01 | 30.24±0.04 | 46±0.01 | 35.13±0.07 | N.D. | 51.82±0.03 | N.D. | 27.42±0.04 | 217.97±0.05 | 69.62±0.02 |
| **50** | 5.11±0.04 | 10.29±0.09 | 88.66±0.02 | 7.8±0.01 | N.D. | 33.88±0.08 | 139.37±0.54 | 1.46±0 | 143.91±0.07 | 66.57±0.03 |

N.D.: not detected.


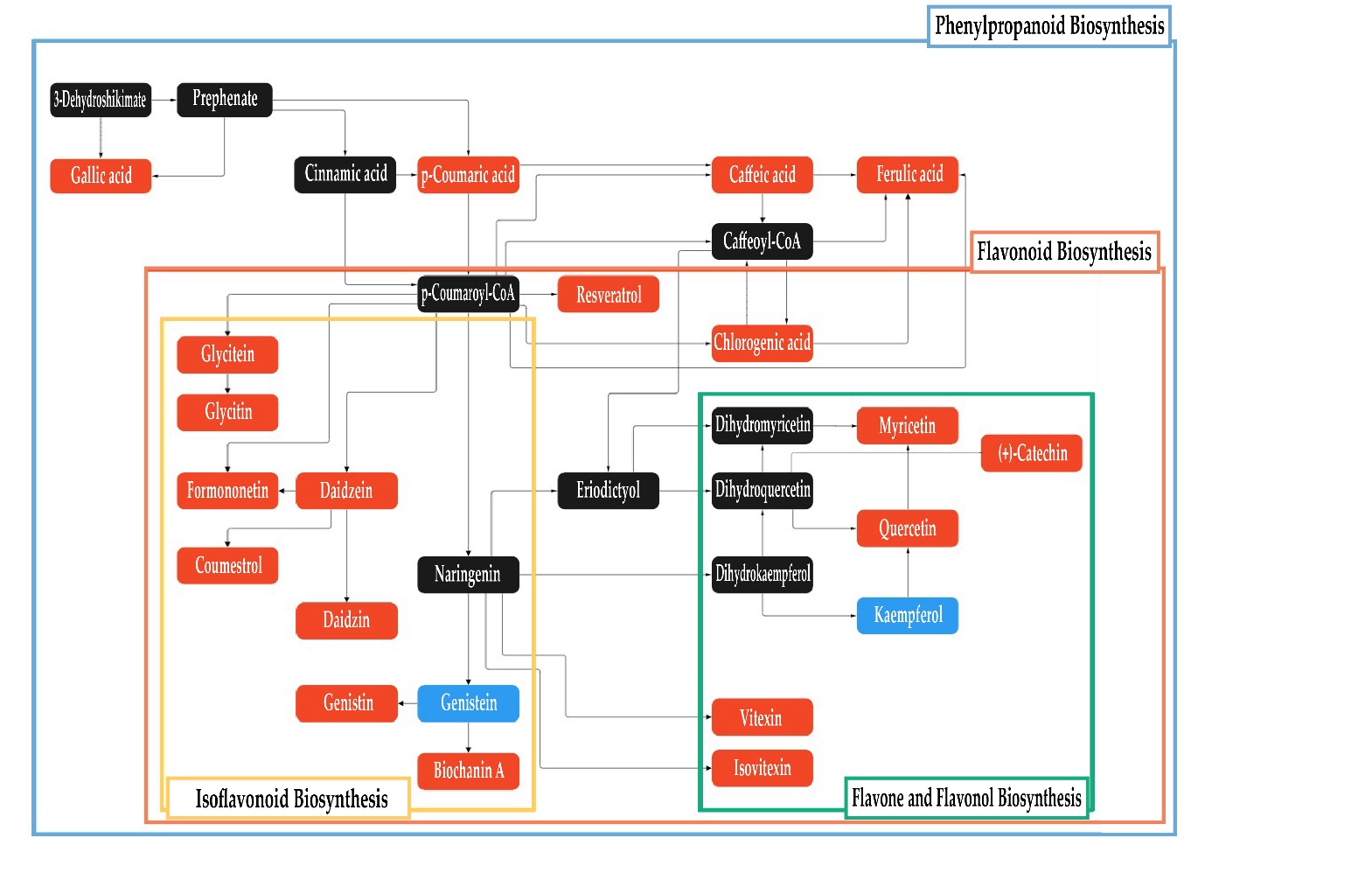


**Supplementary figure 2.** Biosynthetic pathway of the secondary metabolites in mungbean sprout. The metabolites used as standard compounds for UPLC are indicates by red and blue. Red and blue indicates the compounds detected and undetected in mungbean sprouts extracts, respectively.

**Supplementary table 5.** Correlation of the contents of secondary metabolites among 12 selected genotypes.

|  | **Catechin** | **Daidzin** | **Ferulic acid** | **Caffeic acid** | **p-Coumaric acid** | **Formononetin** | **Vitexin** | **Isovitexin** | **Daidzein** | **Glycitein** | **Myricetin** | **Neochlorogenic acid** | **Chlorogenic acid** |
| --- | --- | --- | --- | --- | --- | --- | --- | --- | --- | --- | --- | --- | --- |
| **Catechin** | 1.00 |  |  |  |  |  |  |  |  |  |  |  |  |
| **Daidzin** | -0.36 | 1.00 |  |  |  |  |  |  |  |  |  |  |  |
| **Ferulic acid** | 0.13 | 0.19 | 1.00 |  |  |  |  |  |  |  |  |  |  |
| **Caffeic acid** | -0.44 | 0.57 | 0.20 | 1.00 |  |  |  |  |  |  |  |  |  |
| **p-Coumaric acid** | -0.31 | 0.27 | 0.01 | 0.10 | 1.00 |  |  |  |  |  |  |  |  |
| **Formononetin** | 0.72 | -0.55 | 0.04 | -0.12 | -0.31 | 1.00 |  |  |  |  |  |  |  |
| **Vitexin** | 0.74 | -0.47 | -0.11 | -0.83 | -0.24 | 0.27 | 1.00 |  |  |  |  |  |  |
| **Isovitexin** | 0.39 | 0.23 | 0.13 | -0.41 | 0.29 | 0.04 | 0.43 | 1.00 |  |  |  |  |  |
| **Daidzein** | 0.46 | 0.27 | -0.36 | -0.22 | -0.04 | 0.23 | 0.41 | 0.65 | 1.00 |  |  |  |  |
| **Glycitein** | 0.67 | -0.61 | 0.16 | -0.22 | -0.30 | 0.94 | 0.32 | 0.09 | 0.13 | 1.00 |  |  |  |
| **Myricetin** | 0.44 | 0.38 | 0.00 | 0.16 | 0.23 | 0.16 | 0.25 | 0.54 | 0.61 | 0.13 | 1.00 |  |  |
| **Neochlorogenic acid** | 0.88 | -0.35 | -0.08 | -0.25 | -0.07 | 0.79 | 0.50 | 0.34 | 0.50 | 0.68 | 0.50 | 1.00 |  |
| **Chlorogenic acid** | 0.47 | -0.19 | -0.34 | -0.22 | 0.47 | 0.36 | 0.39 | 0.39 | 0.49 | 0.29 | 0.61 | 0.73 | 1.00 |
